# Narrow bounds for the quantum capacity of thermal attenuators

**DOI:** 10.1038/s41467-018-06848-0

**Published:** 2018-10-18

**Authors:** Matteo Rosati, Andrea Mari, Vittorio Giovannetti

**Affiliations:** 1 Física Teòrica: Informació i Fenòmens Quàntics, Departament de Física, Universitat Autònoma de Barcelona, 08193 Bellaterra, Spain; 2 NEST, Scuola Normale Superiore and Istituto Nanoscienze-CNR, 56127 Pisa, Italy

## Abstract

Thermal attenuator channels model the decoherence of quantum systems interacting with a thermal bath, e.g., a two-level system subject to thermal noise and an electromagnetic signal traveling through a fiber or in free-space. Hence determining the quantum capacity of these channels is an outstanding open problem for quantum computation and communication. Here we derive several upper bounds on the quantum capacity of qubit and bosonic thermal attenuators. We introduce an extended version of such channels which is degradable and hence has a single-letter quantum capacity, bounding that of the original thermal attenuators. Another bound for bosonic attenuators is given by the bottleneck inequality applied to a particular channel decomposition. With respect to previously known bounds we report better results in a broad range of attenuation and noise: we can now approximate the quantum capacity up to a negligible uncertainty for most practical applications, e.g., for low thermal noise.

## Abstract (web summary)

Bounding the capacity of thermal attenuators would give a powerful instrument to describe decoherence occurring in optical fibres and free space links. Here, the authors improve on the existing upper bounds in the region of small thermal noise, which is of interest for quantum communication.

## Introduction

The study of information transmission between distant parties has attracted much theoretical attention since the seminal work of Shannon^1,2^, who gave birth to the field of information theory by determining the ultimate limits for compression and transmission rate. The latter is called information capacity and it depends on the specific channel that is used to model a physical transmission process. Hence, since the information carriers are ultimately governed by the laws of quantum physics, in more recent years there has been interest in analyzing the communication problem in a quantum setting, giving birth to the field of quantum communication and information theory^3–7^. Several results have been obtained so far, such as: an expression for the capacity of a quantum channel for the transmission of classical information^8–12^ and its explicit value for some classes of channels^5,6,13–19^; the use of entanglement^20^ as a resource for communication^21,22^; the capacity of a quantum channel for the transmission of quantum information, i.e., of states preserving quantum coherence^23–26^. The latter is called quantum capacity of the channel and constitutes the main focus of this work. As usual in information theory, although a formula exists for this quantity, it is quite difficult to compute for general channels, due for example to the necessary regularization that takes into account the use of entangled inputs over multiple channel uses^27^. The problem simplifies considerably for the so called degradable channels^19,28–31^, but it also exhibits some striking features in other cases^32–36^. An important class of channels is that of Thermal Attenuators (TAs), which describe the effect of energy loss due to the interaction with a thermal environment. Common examples of this class are the qubit thermal attenuator or generalized amplitude damping channel^7,19^ and its infinite-dimensional counterpart given by the bosonic Gaussian thermal attenuator^3,4,37^. Physically, the former is a good model for the thermalization process of a two-level system (e.g., a single-qubit quantum memory) in contact with a thermal bath, while the latter is the standard description for optical-fiber and free-space communications in the presence of thermal noise. Notice that thermal noise at room temperature is not negligible for low-frequency electromagnetic signals like, e.g., infrared lasers, microwaves, radio waves, etc. For example, a crude estimate of the thermal noise in a real communication channel can be obtained by using the Bose–Einstein distribution at the desired wavelength to estimate the excess contribution to the mean photon number of the transmitted signal: this grows from *O*(10^−14^) at telecom wavelengths, i.e., 1550 nm, and *O*(10) at microwave wavelengths, i.e., 1 mm and above. Accordingly, although the thermal noise may be negligible at telecom wavelengths, it becomes increasingly important as one spans the whole optical domain and it is a crucial parameter in the microwave domain. Hence the study of information transmission on TAs is of particular relevance for future quantum communication networks where hybrid interfaces will be employed, e.g., superconducting qubits connected by microwave communication lines, see refs. ^38,39^. In both the qubit and bosonic cases, the corresponding quantum capacity is known^19,40^ only for a zero-temperature environment, since in this limit both channels are degradable^28–30^. However the more general finite-temperature case breaks the degradability property and it has not been successfully tackled so far, apart from establishing some upper and lower bounds^41–43^.

In this article, we introduce a general method to compute new bounds on the capacity of any quantum channel which is weakly degradable in the sense of ref. ^29,31^. It is based on the purification of the environment by an additional system, which is then included in the output of an extended version of the original channel. Degradability is restored for such extended channel and its quantum capacity can be easily computed, providing an upper bound on the capacity of the original channel, which is tight in the limit of small environmental noise. This result applies to any weakly degradable channel but, in order to be more explicit, here we compute the associated upper bound for both the qubit and continuous-variable thermal attenuators. Moreover, for the Gaussian attenuator, we provide an additional bound based on the bottleneck inequality applied to a twisted version of the channel decomposition used in^15,31,44^. Eventually we compare all our bounds with those already known in the literature and in particular with the recent results of Pirandola et al.^42^ and Sharma et al.^43^ We show a significant improvement with respect to the state-of-the-art for a large region of attenuation and noise parameters, shrinking the unknown value of the quantum capacity within an error bar which is so narrow to be irrelevant for many practical applications.

## Results

### Thermal attenuator channels

Let us consider a communication channel that connects two parties. The sender, Alice, wants to transmit the quantum state $\hat \rho _{\mathrm{A}} \in {\frak S}\left( {{\cal H}_{\mathrm{A}}} \right)$, represented by a positive density operator of unit trace on the Hilbert space of the system, ${\cal H}_{\mathrm{A}}$. At the other end of the channel Bob receives a transformed quantum state on the Hilbert space ${\cal H}_{\mathrm{B}}$. Any physical transformation applied to the state during the transmission can be represented by a quantum channel Φ, i.e., a linear, completely-positive and trace-preserving map, which evolves the initial state as $\hat \rho _{\mathrm{B}} = {\mathrm{\Phi }}\left( {\hat \rho _{\mathrm{A}}} \right)$.

**Fig. 1:**
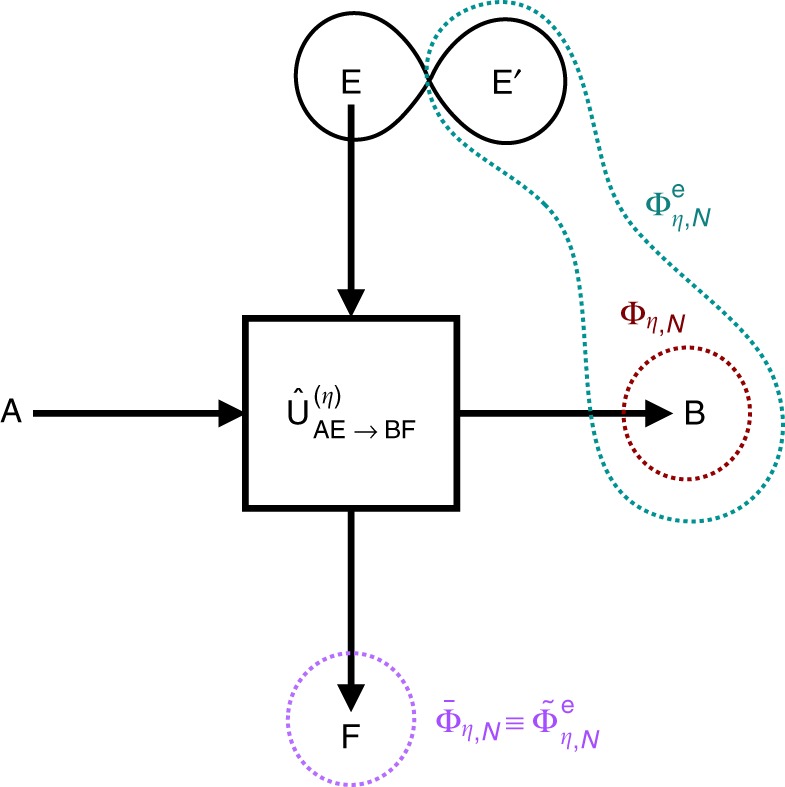
Unitary representation of a thermal attenuator channel, its extended channel and their complementaries. The system A interacts with the environment E, which is in a thermal state of mean energy *N*, via a linear coupling, $\hat U_{{\mathrm{AE}} \to {\mathrm{BF}}}^{(\eta )}$. The parameter 1−*η* determines the fraction of energy dispersed from the system into the environment, while the total energy is preserved. The output of the channel Φ_*η*,*N*_ is recovered by discarding the output environment F, while that of its weak complementary ${\bar{\mathrm \Phi }}_{\eta ,N}$ by discarding the output system B. The two channels are called weakly complementary to each other because E is in a mixed state and hence this unitary representation is not a proper Stinespring dilation. By purifying the input environment via an ancilla E′, entangled with E, we obtain the extended channel ${\mathrm{\Phi }}_{\eta ,N}^{\mathrm{e}}$ with output BE’ and its strong complementary ${\tilde{\mathrm \Phi }}_{\eta ,N}^{\mathrm{e}}$ with output F. Note that the latter coincides with ${\bar{\mathrm \Phi }}_{\eta ,N}$

The class of channels studied in this article is that of thermal attenuators, Φ_*η*,*N*_, originating from the following physical representation (see Fig. 1). An energy-preserving interaction $\hat U_{{\mathrm{AE}} \to {\mathrm{BF}}}^{(\eta )}$, parametrized by a transmissivity parameter *η* ∈ [0, 1], couples the input system $\hat \rho _{\mathrm{A}}$ with an environment described by a thermal state $\hat \tau _{\mathrm{E}} \propto {\mathrm{exp}}\left( { - \hat H_{\mathrm{E}}} \right)$, where $\hat H_{\mathrm{E}}$ is the bath hamiltonian, of dimension equal to that of the system, and the state has mean energy $N = {\mathrm{Tr}}\left[ {\hat H_{\mathrm{E}}\hat \tau _{\mathrm{E}}} \right] \ge 0$ in dimensionless units. The total output state can be written as

1 $$\hat \rho _{{\mathrm{BF}}} = \hat U_{{\mathrm{AE}} \to {\mathrm{BF}}}^{(\eta )}\left( {\hat \rho _{\mathrm{A}} \otimes \hat \tau _{\mathrm{E}}} \right)\hat U_{{\mathrm{AE}} \to {\mathrm{BF}}}^{(\eta )\dagger },$$

while the action of the channel is obtained by tracing out the output environmental system F:

2 $${\mathrm{\Phi }}_{\eta ,N}\left( {\hat \rho _{\mathrm{A}}} \right) = {\mathrm{Tr}}_{\mathrm{F}}\left[ {\hat \rho _{{\mathrm{BF}}}} \right].$$

This general framework is particularly relevant in the two paradigmatic cases in which the system is represented by a two-level system or by a single bosonic mode. Both situations are quite important for practical applications since they model the effect of damping and thermal noise on common information carriers, typically used in experimental implementations of quantum information and quantum communication protocols. In the following, we introduce in detail these two kinds of physical systems.

In order to describe a two-level system, we fix as a basis $\left| 0 \right\rangle$ and $\left| 1 \right\rangle$ corresponding to the ground and excited states respectively. In this basis we can represent a generally mixed quantum state with a density matrix:

3 $$\hat \rho = \left[ {\begin{array}{*{20}{c}} {1 - p} & \gamma \\ {\gamma ^ \ast } & p \end{array}} \right],$$

where *p* ∈ [0, 1] is the mean population of the excited state and $\gamma \in {\Bbb C}$ is a complex coherence term, with $\left| \gamma \right|^2 \le p(1 - p)$. The thermal attenuator channel ${\mathrm{\Gamma }}_{\eta ,{\cal N}}$ with *η* ∈ [0, 1] and *N* ∈ [0, 1/2], also known in the literature as generalized amplitude damping channel^7,19^, acts on the matrix elements in the following way:

4 $$p\mathop{\longrightarrow}\limits^{{{\mathrm{\Gamma }}_{\eta ,N}}}p\prime = \eta p + (1 - \eta )N,$$

5 $$\gamma \mathop{\longrightarrow}\limits^{{{\mathrm{\Gamma }}_{\eta ,N}}}\gamma \prime = \sqrt \eta \gamma ,$$

and admits a representation according to the general structure given in Eqs. (1) and (2). In this case, the environment is given by a thermal two-level system:

6 $$\hat \tau = \left[ {\begin{array}{*{20}{c}} {1 - N} & 0 \\ 0 & N \end{array}} \right],$$

where *N* ∈ [0, 1/2] represents the dimensionless mean energy and the bath hamiltonian is $\hat H_{\mathrm{E}} \propto \left| 1 \right\rangle \left\langle 1 \right|$. The unitary interaction is instead given by an energy-preserving rotation on the subspace $\left\{ {\left| {01} \right\rangle ,\left| {10} \right\rangle } \right\}$ of the joint Hilbert space of the system:

7 $$\hat U^{(\eta )} = \left[ {\begin{array}{*{20}{c}} 1 & 0 & 0 & 0 \\ 0 & {\sqrt \eta } & {\sqrt {1 - \eta } } & 0 \\ 0 & { - \sqrt {1 - \eta } } & {\sqrt \eta } & 0 \\ 0 & 0 & 0 & 1 \end{array}} \right],$$

physically inducing the hopping of excitations between the system and the environment. It is easy to check that, tracing out the environmental two-level system, we obtain the single qubit thermal attenuator channel defined in (4) and (5).

A single mode of electromagnetic radiation instead is formally equivalent to an infinite-dimensional quantum harmonic oscillator. It can be described in terms of bosonic annihilation and creation operators $\hat a$ and $\hat a^\dagger$, obeying the bosonic commutation relation $\left[ {\hat a,\hat a^\dagger } \right] = 1$ or, equivalently, in terms of the quadratures $\hat q = \left( {\hat a + \hat a^\dagger } \right){\mathrm{/}}\sqrt 2$ and $\hat p = i\left( {\hat a^\dagger - \hat a} \right){\mathrm{/}}\sqrt 2$, such that $\left[ {\hat q,\hat p} \right] = i$. Given *n* modes and introducing the vector of quadrature operators ${\hat{\boldsymbol r}} = \left( {\hat q_1,\hat p_1, \ldots ,\hat q_n,\hat p_n} \right)^\intercal$, one can define the characteristic function^37,45^ of a bosonic state $\hat \rho$:

8 $$\chi \left( {\boldsymbol{\xi }} \right) = {\mathrm{Tr}}\left[ {\hat \rho \,e^{{\boldsymbol{\xi }}^T{\mathrm{\Omega }}^T{\hat{\boldsymbol r}}}} \right],$$

where ${\bf{\xi }} \in {\Bbb R}^{2n}$ and $${\mathrm{\Omega }} = \left[ {\begin{array}{*{20}{c}} 0 & 1 \\ { - 1} & 0 \end{array}} \right]^{ \oplus n}$$ is the symplectic form. The most common bosonic states are Gaussian states, i.e. those whose characteristic function is Gaussian:

9 $$\chi _{\mathrm{G}}\left( {\boldsymbol{\xi }} \right) = {\mathrm{exp}}\left\{ { - \frac{1}{4}{\boldsymbol{\xi }}^T{\mathrm{\Omega }}^TV{\mathrm{\Omega }}\,{\boldsymbol{\xi }} + {\boldsymbol{\xi }}^T{\mathrm{\Omega }}^T{\boldsymbol{m}}} \right\},$$

uniquely determined by the first and second moments of the state $\hat \rho _{\mathrm{G}}$, given respectively by the vector of mean values ***m*** and by the symmetric covariance matrix *V* of the quadrature operators. The single-mode thermal attenuator channel ${\cal E}_{\eta ,N}$ has been extensively studied in the context of Gaussian quantum information^3,27,37^ and its action on the first and second moments is given by:

10 $${\boldsymbol{m}}\mathop{\longrightarrow}\limits^{{{\cal E}\eta ,N}}{\boldsymbol{m}}\prime = \sqrt \eta {\boldsymbol{m}},$$

11 $$V\mathop{\longrightarrow}\limits^{{{\cal E}\eta ,N}}V\prime = \eta V + (1 - \eta )(2N + 1){\mathbf{1}}_2,$$

where *η* ∈ [0, 1] and *N* ≥ 0. As for the qubit case, also this infinite-dimensional channel can be represented using the general picture of Eqs. (1) and (2). Indeed, introducing the single-mode Gaussian thermal state $\hat \tau$ characterized by ${\boldsymbol{m}}_{\hat \tau } = 0$ and $V_{\hat \tau } = (2N + 1){\mathbf{1}}$, the thermal attenuator channel can be generated by a passive unitary interaction $\hat U$ that acts on the bosonic operators of the system, $\hat a$, and of the environment, $\hat a_{\mathrm{E}}$, as a beam splitter:

12 $$U^\dagger \hat aU = \sqrt \eta \hat a + \sqrt {1 - \eta } \hat a_{\mathrm{E}},$$

13 $$U^\dagger \hat a_{\mathrm{E}}U = - \sqrt {1 - \eta } \hat a + \sqrt \eta \hat a_{\mathrm{E}}.$$

It is easy to check that, tracing out the environmental mode, one recovers the definition of the single-mode thermal attenuator given in Eqs. (10) and (11).

### Known bounds for the quantum capacity of thermal attenuators

The quantum capacity *Q*(Φ) of a channel Φ is defined as the maximum rate at which quantum information can be transferred by using the channel *N* times with vanishing error in the limit *N* → ∞. It is well known^23–26^ that this quantity can be expressed as:

14 $$Q({\mathrm{\Phi }}) = \mathop {{{\mathrm{lim}}}}\limits_{N \to \infty } \frac{1}{N}\mathop {{{\mathrm{max}}}}\limits_{\hat \rho \in {\cal G}\left( {{\cal H}_{\mathrm{A}}^{ \otimes N}} \right)} J\left( {\hat \rho ,{\mathrm{\Phi }}^{ \otimes N}} \right),$$

where

15 $$J\left( {\hat \rho ,{\mathrm{\Phi }}} \right) = S({\mathrm{\Phi }}(\hat \rho )) - S({\tilde{\mathrm \Phi }}(\hat \rho ))$$

is the coherent information^27,46^ and $S(\hat \rho ) = - {\mathrm{tr}}\left\{ {\hat \rho \,{\mathrm{log}}_2\hat \rho } \right\}$ is the Von Neumann entropy; note that all logarithms henceforth are understood as base-2. Finally, $\tilde \Phi _{}^{}$ is the complementary channel^28^, obtained from the Stinespring dilation of Φ by tracing out the system instead of the environment, as detailed in the next subsection. Because of the peculiar superadditivity phenomenon^32–36^, the so called single-letter capacity

16 $$Q_1({\mathrm{\Phi }}) = \mathop {{{\mathrm{max}}}}\limits_{\hat \rho \in {\cal H}_{\mathrm{A}}} J(\hat \rho ,{\mathrm{\Phi }})$$

is in general smaller than the actual capacity of the channel.

This fact directly gives a lower bound for the quantum capacity of the qubit thermal attenuator:

17 $$Q\left( {{\mathrm{\Gamma }}_{\eta ,N}} \right) \ge Q_1\left( {{\mathrm{\Gamma }}_{\eta ,N}} \right) = \mathop {{{\mathrm{max}}}}\limits_p J\left( {\hat \rho = \left[ {\begin{array}{*{20}{c}} {1 - p} & 0 \\ 0 & p \end{array}} \right],{\mathrm{\Gamma }}_{\eta ,N}} \right)$$

where the maximization is only with respect to *p* ∈ [0, 1], since one can check numerically that for this channel optimal states are diagonal; this quantity can be easily numerically computed for all values of *η* and *N*. Similarly, a lower bound can be obtained also for the bosonic counterpart of the thermal attenuator by restricting the optimization over the class of Gaussian input states^41^:

18 $$\begin{array}{*{20}{l}} {Q\left( {{\cal E}_{\eta ,N}} \right)} \hfill & \ge \hfill & {Q_1\left( {{\cal E}_{\eta ,N}} \right) \ge \mathop {{{\mathrm{max}}}}\limits_{\hat \rho _{\mathrm{G}}} J\left( {\hat \rho _{\mathrm{G}},{\cal E}_{\eta ,N}} \right)} \hfill \\ {} \hfill & = \hfill & {{\mathrm{max}}\left\{ {0,{\mathrm{log}}_2\frac{\eta }{{1 - \eta }} - g(N)} \right\},} \hfill \end{array}$$

where *ρ*_G_ varies over the set Gaussian states and $g(N)$ = $(N + 1){\mathrm{log}}_2(N + 1) - N\,{\mathrm{log}}_2N$ corresponds to the entropy of the thermal state of the environment.

Whether the right-hand-sides of (17) and (18) are equal or not to the true quantum capacity of the associated channels is still an important open problem in quantum information. It can be shown^19,28,40^, that for a zero-temperature environment this is the case, i.e., for *N* = 0 all inequalities in (17) and (18) are saturated, giving the quantum capacity of both the qubit and the Gaussian attenuators. For *N* > 0 instead, the capacity is still unknown, apart from some upper bounds. For the qubit case, we are not aware of any upper bounds proposed in the literature, while for the Gaussian thermal attenuator the best bounds at the moment are those recently introduced in refs. ^42,43^, which can be combined to get the following expression:

19 $$\begin{array}{l}Q\left( {{\cal E}_{\eta ,N}} \right) \le {\mathrm{min}}\left\{ {Q_{{\mathrm{PLOB}}}\left( {{\cal E}_{\eta ,N}} \right),Q_{{\mathrm{SWAT}}}\left( {{\cal E}_{\eta ,N}} \right)} \right\},\\ Q_{{\mathrm{PLOB}}}\left( {{\cal E}_{\eta ,N}} \right) = {\mathrm{max}}\left\{ {0, - {\mathrm{log}}_2\left[ {(1 - \eta )\eta ^N} \right] - g(N)} \right\},\\ Q_{{\mathrm{SWAT}}}\left( {{\cal E}_{\eta ,N}} \right) = {\mathrm{max}}\left\{ {0,{\mathrm{log}}_2[\eta {\mathrm{/}}(1 - \eta )] - {\mathrm{log}}_2[N + 1]} \right\}.\end{array}$$

Let us note that *Q*_PLOB_ is actually a bound on the quantum capacity assisted by two-way classical communication^42^ and thus trivially bounds also the simpler unassisted capacity discussed in this article (strong-converse bounds for the two-way capacity were derived, e.g., in refs. ^47,48^). The other bound instead, *Q*_SWAT_, is itself a bound on the unassisted capacity and it has been shown^43^ to beat other possible bounds based on $\epsilon$-degradability^49^. In the next sections we are going to derive new upper bounds which are significantly closer to the lower limits (17) and (18), especially in the low temperature regime.

### The extended channel

In this subsection we first review the notions of degradability and weak degradability and then we introduce an extended version of thermal attenuator maps, whose quantum capacity is easier to compute and can be used as a useful upper bound.

Let us come back to the description of a generic attenuator map Φ_*η*,*N*_, valid both for qubit and bosonic systems, that we have previously defined in Eqs. (1) and (2). If, instead of tracing out the environment as done in Eq. (2), we trace out the system, we get what has been defined in refs. ^29,31^ as the weakly complementary channel:

20 $${\bar{\mathrm \Phi }}_{\eta ,N}\left( {\hat \rho _{\mathrm{A}}} \right) = {\mathrm{Tr}}_{\mathrm{B}}\left[ {\hat \rho _{{\mathrm{BF}}}} \right],$$

physically representing the flow of information from the system into the environment. Notice that this is different form the standard notion of complementary channel:

21 $${\tilde{\mathrm \Phi }}_{\eta ,N}\left( {\hat \rho _{\mathrm{A}}} \right) = {\mathrm{Tr}}_{\mathrm{B}}\left[ {\hat \rho _{{\mathrm{BFE}}\prime }} \right],$$

where

22 $$\hat \rho _{{\mathrm{BFE}}\prime } = \hat U_{{\mathrm{AE}} \to {\mathrm{BF}}}^{(\eta )}\left( {\hat \rho _{\mathrm{A}} \otimes \left| \tau \right\rangle \left\langle \tau \right|_{{\mathrm{EE}}\prime }} \right)\hat U_{{\mathrm{AE}} \to {\mathrm{BF}}}^{(\eta )\dagger }$$

and $\left| \tau \right\rangle _{{\mathrm{EE}}\prime }$ is a purification of the environment, i.e.,$\hat \tau _{\mathrm{E}} = {\mathrm{Tr}}_{{\mathrm{E}}\prime }\left[ {\left| \tau \right\rangle \left\langle \tau \right|_{{\mathrm{EE}}\prime }} \right]$. The weak and standard complementary channels become equivalent (up to a trivial isometry) only in the particular case in which the environment is initially pure (zero-temperature limit). Finally, we also remark that the two different types of complementarity induce different definitions of degradability. A generic channel Φ is degradable^28,30^, if there exists another quantum channel Δ such that ${\tilde{\mathrm \Phi }} = {\mathrm{\Delta }} \circ {\mathrm{\Phi }}$. Similarly, a generic channel Φ is weakly degradable^29,31^, if there exists another quantum channel Δ such that ${\bar{\mathrm \Phi }} = {\mathrm{\Delta }} \circ {\mathrm{\Phi }}$. For degradable channels, the capacity is additive and much easier to determine. Unfortunately, typical models of quantum attenuators, as the qubit and the bosonic examples considered in this work, are degradable only for *N* = 0 but become only weakly degradable for *N* > 0 and this is the main reason behind the hardness in computing their quantum capacity.

In order to circumvent this problem, we define the extended version of a thermal attenuator channel as

23 $${\mathrm{\Phi }}_{\eta ,N}^{\mathrm{e}}\left( {\hat \rho _{\mathrm{A}}} \right) = {\mathrm{Tr}}_{\mathrm{F}}\left[ {\hat \rho _{{\mathrm{BFE}}\prime }} \right],$$

where $\hat \rho _{{\mathrm{BFE}}\prime }$ is the global state defined in (22). In other words, ${\mathrm{\Phi }}_{\eta ,N}^{\mathrm{e}}$ represents a situation in which Bob has access not only to the output system B but also to the purifying part E′ of the environment, see Fig. 1. A remarkable fact is that, locally, the auxiliary system E′ remains always in the initial thermal state because it is unaffected by the dynamics; however, E′ can be correlated with B and this fact can be exploited by Bob to retrieve more quantum information. In general, since trowing away E′ can only reduce the quantum capacity, one always has $Q\left( {{\mathrm{\Phi }}_{\eta ,N}} \right) \le Q\left( {{\mathrm{\Phi }}_{\eta ,N}^{\mathrm{e}}} \right)$.

The advantage of dealing with the extended channel ${\mathrm{\Phi }}_{\eta ,N}^{\mathrm{e}}$ is that it is degradable whenever the original channel Φ_*η*,*N*_ is weakly degradable. This fact follows straightforwardly from the observation that the complementary channel of ${\mathrm{\Phi }}_{\eta ,N}^{\mathrm{e}}$ is the weakly complementary channel of Φ_*η*,*N*_, i.e.,

24 $${\tilde{\mathrm \Phi }}_{\eta ,N}^e\left( {\hat \rho _A} \right) = {\mathrm{Tr}}_{{\mathrm{BE}}\prime }\left[ {\hat \rho _{{\mathrm{BFE}}\prime }} \right] = {\bar{\mathrm \Phi }}_{\eta ,N}\left( {\hat \rho _A} \right).$$

The degradability of ${\mathrm{\Phi }}_{\eta ,N}^{\mathrm{e}}$ significantly simplifies the evaluation of $Q\left( {{\mathrm{\Phi }}_{\eta ,N}^{\mathrm{e}}} \right)$ and provides a very useful upper bound for the quantum capacity of thermal attenuators:

25 $$Q\left( {{\mathrm{\Phi }}_{\eta ,N}} \right) \le Q\left( {{\mathrm{\Phi }}_{\eta ,N}^{\mathrm{e}}} \right) = Q_1\left( {{\mathrm{\Phi }}_{\eta ,N}^{\mathrm{e}}} \right),$$

where in the last step we used the additivity property valid for all degradable channels^28,30^.

Another useful property of the extended channel, which follows from Eq. (24) and the definition of coherent information, Eq. (15), is the following:

26 $$J\left( {\rho ,{\mathrm{\Phi }}_{\eta ,N}^{\mathrm{e}}} \right) = - J\left( {\rho ,{\bar{\mathrm \Phi }}_{\eta ,N}} \right).$$

relating the coherent information of the extended and of the weakly complementary channels.

Below we compute more explicitly the previous bound (25) for the specific cases of discrete- and continuous-variable thermal attenuators.

In the case of two-level systems, the purification of the thermal state given in Eq. (6) is

27 $$\left| \tau \right\rangle = \sqrt {1 - N} \left| {00} \right\rangle + \sqrt N \left| {11} \right\rangle .$$

**Fig. 2:**
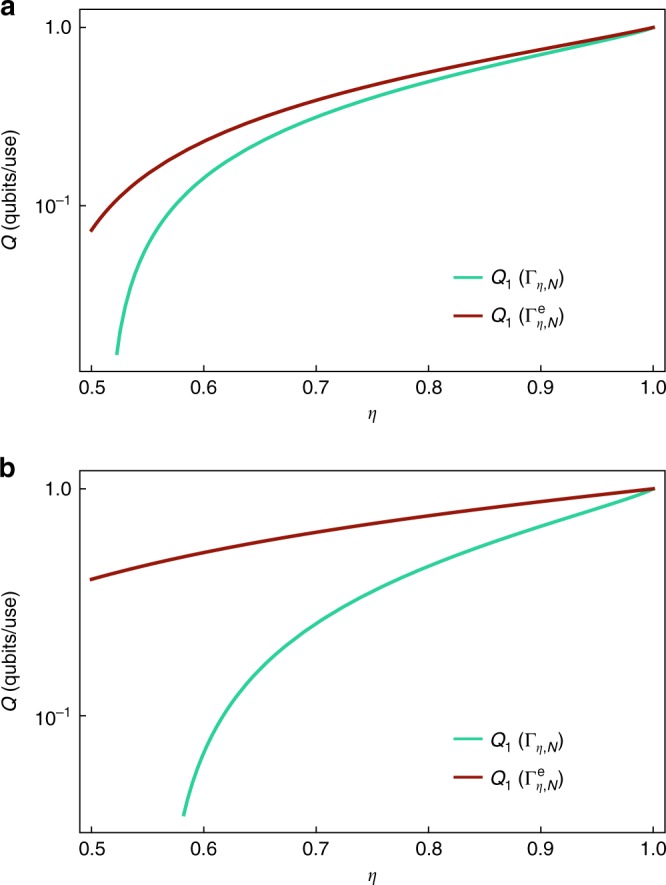
Bounds of the quantum capacity of a qubit thermal attenuator. Plot (log-linear scale) of the lower bound *Q*_1_(Γ_*η*,*N*_) (green line), Eq. (17), and upper bound $Q_1\left( {{\mathrm{\Gamma }}_{\eta ,N}^{\mathrm{e}}} \right)$ (red line), Eq. (28), of the quantum capacity of the qubit thermal attenuator channel as a function of the attenuation parameter *η* for two values of noise: **a** *N* = 0.01, **b** *N* = 0.1. The lower bound is given by the single-letter capacity of the channel, whereas the upper bound by the capacity of the extended channel, which equals its single-letter expression because of degradability. The two bounds are close for small noise and weak attenuation

The channel Γ_*η*,*N*_ can be weakly degraded to ${\bar{\mathrm \Gamma }}_{\eta ,N}$ by the composite map Δ = Ψ_*μ*_${\circ}$ Γ_*η*′, *N*_, where *η*′ = (1 − *η*)*/η*. Here *Ψ*_*μ*_ is a phase-damping channel^7^ of parameter *μ* = 1 − 2*N*, which acts on the generic qubit state of Eq. (3) by damping the coherence matrix element as $\gamma \mapsto \mu \gamma$ while leaving the population *p* constant. Hence, the extended channel ${\mathrm{\Gamma }}_{\eta ,N}^{\mathrm{e}}$ defined as in (23) is degradable and from (25) we get

28 $$Q\left( {{\mathrm{\Gamma }}_{\eta ,N}} \right) \le Q_1\left( {{\mathrm{\Gamma }}_{\eta ,N}^{\mathrm{e}}} \right) = \mathop {{{\mathrm{max}}}}\limits_p J\left( {\hat \rho = \left[ {\begin{array}{*{20}{c}} {1 - p} & 0 \\ 0 & p \end{array}} \right],{\mathrm{\Gamma }}_{\eta ,N}^{\mathrm{e}}} \right),$$

where the optimization over the single parameter *p* can be efficiently performed numerically for all values of *η* and *N*, giving the result presented in Fig. 2. In this case, the fact that we can reduce the optimization over diagonal input states follows from the symmetry of the coherent information under the matrix-element flipping *γ* → −*γ* and from the concavity of the coherent information for degradable channels^50^.

We note that, by construction, the gap between the lower (17) and the upper (28) bounds closes in the limit *N* → 0, where we recover the zero-temperature capacity of the amplitude damping channel consistently with^19,41^.

In the case of bosonic systems instead, the purification of the thermal environmental mode $\hat \tau$ with first moments ${\boldsymbol{m}}_{\hat \tau } = 0$ and covariance matrix $V_{\hat \tau } = (2N + 1){\mathbf{1}}$ is a Gaussian two-mode squeezed state $\left| \tau \right\rangle$^37^, characterized by ${\boldsymbol{m}}_{|\tau \rangle } = 0$ and

29 $$V_{|\tau \rangle } = \left( {\begin{array}{*{20}{c}} {V_{\hat \tau }} & {\sigma _3\sqrt {V_{\hat \tau }^2 - {\mathbf{1}}_2} } \\ {\sigma _3\sqrt {V_{\hat \tau }^2 - {\mathbf{1}}_2} } & {V_{\hat \tau }} \end{array}} \right),$$

where *σ*_3_ = diag(1, −1) is the third Pauli matrix. The thermal attenuator ${\cal E}_{\eta ,N}$ is weakly degradable^29,31^, since its weakly complementary channel can be expressed as $\bar {\cal E}_{\eta ,N} = {\mathrm{\Delta }} \circ {\cal E}_{\eta ,N}$ where ${\mathrm{\Delta }} = {\cal E}_{\eta \prime ,N}$, with *η*′ = (1 − *η*)/*η*. Hence, ${\cal E}_{\eta ,N}^{\mathrm{e}}$ is degradable and we can apply the general upper bound (25). Moreover, as shown in^40,51^, the quantum capacity of a degradable Gaussian channel is maximized by Gaussian states with fixed second moments, so that we can write

30 $$Q\left( {{\cal E}_{\eta ,N}} \right) \le Q_1\left( {{\cal E}_{\eta ,N}^{\mathrm{e}}} \right) = \mathop {{{\mathrm{max}}}}\limits_{\hat \rho _{\mathrm{G}}} J\left( {\hat \rho _{\mathrm{G}},{\cal E}_{\eta ,N}^{\mathrm{e}}} \right)$$

reducing the problem to a tractable Gaussian optimization. Since the coherent information of the extended channel is concave and symmetric with respect to phase-space rotations and translations, for a fixed energy *n* it is maximized by the thermal state $\hat \tau _n$ with covariance matrix $V_{\hat \tau _n} = (1 + 2n){\mathbf{1}}_2$, see ref. ^52^ Therefore, without energy constraint we have

31 $$Q\left( {{\cal E}_{\eta ,N}} \right) \le Q_1\left( {{\cal E}_{\eta ,N}^{\mathrm{e}}} \right) = \mathop {{{\mathrm{lim}}}}\limits_{n \to \infty } J\left( {\hat \tau _n,{\cal E}_{\eta ,N}^{\mathrm{e}}} \right).$$

**Fig. 3:**
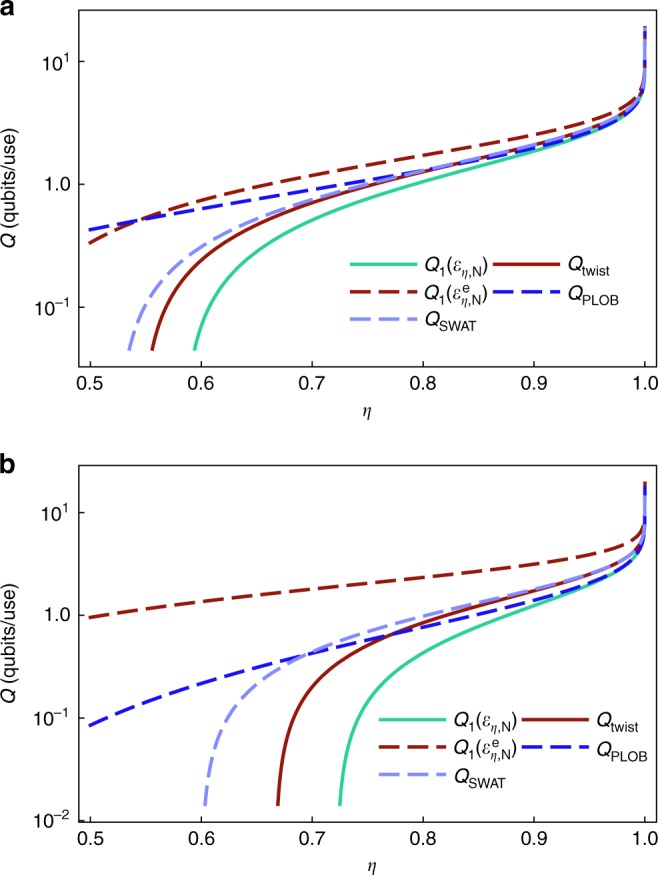
Bounds of the quantum capacity of a bosonic thermal attenuator. Plot (log-linear scale) of the lower bound $Q_1\left( {{\cal E}_{\eta ,N}} \right)$ (green line), Eq. (18), and several upper bounds of the quantum capacity of the bosonic Gaussian thermal attenuator channel as a function of the attenuation parameter *τ* for two values of noise: **a** *N* = 0.1, **b** *N* = 0.5. The upper bounds are given by: the capacity of the extended channel $Q_1\left( {{\cal E}_{\eta ,N}^{\mathrm{e}}} \right)$ (red dashed line), Eq. (33); the capacity of the attenuator channel of the twisted decomposition *Q*_twist_ (red line), Eq. (40); the upper bounds *Q*_PLOB_ of Eq. (19) derived from ref. ^42^ (blue dashed line) and *Q*_SWAT_ of ref. ^43^ (light-blue dashed line). Note that the best upper bound at small noise values is provided by our *Q*_twist_. As the noise increases, *Q*_PLOB_ starts beating the former for weak attenuation, while *Q*_SWAT_ remains always strictly larger than our bound

The last term can be explicitly computed by using the standard formalism of Gaussian states; more simply, we can relate this quantity to the results of ref. ^41^ Indeed, from the property given in Eq. (26), we have

32 $$J\left( {\hat \tau _n,{\cal E}_{\eta ,N}^{\mathrm{e}}} \right) = - J\left( {\hat \tau _n,\bar {\cal E}_{\eta ,N}} \right) = - J\left( {\hat \tau _n,{\cal E}_{1 - \eta ,N}} \right),$$

but the last term is simply the negative of the coherent information of a thermal attenuator of transmissivity *η*′ = 1 − *η* and a thermal input state, a quantity which has been already computed in ref. ^41^ In the limit of *n* → ∞, we get our desired upper bound:

33 $$Q\left( {{\cal E}_{\eta ,N}} \right) \le Q_1\left( {{\cal E}_{\eta ,N}^{\mathrm{e}}} \right) = {\mathrm{max}}\left\{ {0,{\mathrm{log}}_2\frac{\eta }{{1 - \eta }} + g(N)} \right\},$$

where $g(N)$ = $(N + 1){\mathrm{log}}_2(N + 1) - N\,{\mathrm{log}}_2N$, shown in Fig. 3. Comparing the upper bound (33) with the lower bound (18) we observe that we can determine the quantum capacity of the thermal attenuator up to an uncertainty of 2*g*(*N*), which vanishes in the limit of small thermal noise. For the special case *N* = 0, the gap closes and we recover the capacity of the pure lossy channel consistently with the previous results of ref. ^40^

### Twisted decomposition of Gaussian attenuators

In this section, through a completely different method, we derive a bound for the quantum capacity which is tighter than $Q\left( {{\mathrm{\Phi }}_{\eta ,N}^{\mathrm{e}}} \right)$ but applies only to the bosonic version of thermal attenuators.

Let us first introduce a second kind of thermal Gaussian channel: the single-mode amplifier ${\cal A}_{\kappa ,N}$^37^, which combines the input state and the usual thermal state $\hat \tau$ of energy *N* through a two-mode squeezing interaction with gain *κ* > 1. Tracing out the environment, the first and second moments of the quantum state transform in the following way:

34 $${\boldsymbol{m}}\mathop{\longrightarrow}\limits^{{{\cal A}_{\kappa ,N}}}{\boldsymbol{m}}\prime = \sqrt \kappa {\boldsymbol{m}},$$

35 $$V\mathop{\longrightarrow}\limits^{{{\cal A}_{\kappa ,N}}}V\prime = \kappa V + (\kappa - 1)(2N + 1){\mathbf{1}}.$$

In the particular case in which the environment is at zero temperature, i.e., for *N* = 0, the channel ${\cal A}_{\kappa ,0}$ is called quantum-limited amplifier.

It can be shown that all phase-insensitive Gaussian channels can be decomposed as a quantum-limited attenuator followed by a quantum-limited amplifier^31,44^, with important implications for their classical capacity^15–17^. In this work we introduce a twisted version of this decomposition in which the order of the attenuator and of the amplifier is inverted, which is quite useful for bounding the quantum capacity of thermal attenuators. *Lemma 1*. Every thermal attenuator ${\cal E}_{\eta ,N}$ that is not entanglement-breaking can be decomposed as a quantum-limited amplifier followed by a quantum-limited attenuator:

36 $${\cal E}_{\eta ,N} = {\cal E}_{\eta \prime ,0} \circ {\cal A}_{\kappa \prime ,0},$$

with attenuation and gain coefficients given by

37 $$\eta \prime = \eta - N\left( {1 - \eta } \right),\kappa \prime = \eta {\mathrm{/}}\eta \prime$$

The proof can be obtained by direct substitution and using the fact that non-entanglement-breaking attenuators are characterized by the condition *N* < *η*/(1 − *η*)^27,53^ and so both coefficients in (37) are positive and well defined. As shown in the Methods Section, the previous decomposition can be generalized to all phase-insensitive Gaussian channels including thermal amplifiers and additive Gaussian noise channels. It is important to remark that, differently from the decomposition introduced in ref. ^44^ and employed in ref. ^43^, our twisted version does not apply to entanglement-breaking channels. For the purposes of this work, this is not a restriction since all entanglement-breaking channels trivially have zero quantum capacity and we can exclude them from our analysis.

Now, given a thermal attenuator with *N* < *η*/(1 − *η*), we make use of the twisted decomposition (36) obtaining

38 $$Q\left( {{\mathrm{\Phi }}_{\eta ,N}} \right) = Q\left( {{\mathrm{\Phi }}_{\eta \prime ,0} \circ {\cal A}_{\kappa \prime ,0}} \right) \le Q\left( {{\mathrm{\Phi }}_{\eta \prime ,0}} \right)$$

39 $$= {\mathrm{max}}\left\{ {0,{\mathrm{log}}_{\mathrm{2}}\frac{{\eta \prime }}{{1 - \eta \prime }}} \right\},$$

where we used the “bottleneck” inequality *Q*(Φ_1_ ο Φ_2_) ≤ min{*Q*(Φ_1_), *Q*(Φ_2_)} and the exact expression for the capacity of the quantum-limited attenuator^40^. Substituting the value of *η*′ of Eq. (37) into (38), we get our desired upper bound

40 $$Q\left( {{\mathrm{\Phi }}_{\eta ,N}} \right) \le Q_{{\mathrm{twist}}} = {\mathrm{max}}\left\{ {0,{\mathrm{log}}_2\frac{{\eta -N (1 - \eta )}}{{(1 + N)(1 - \eta )}}} \right\}.$$

**Fig. 4:**
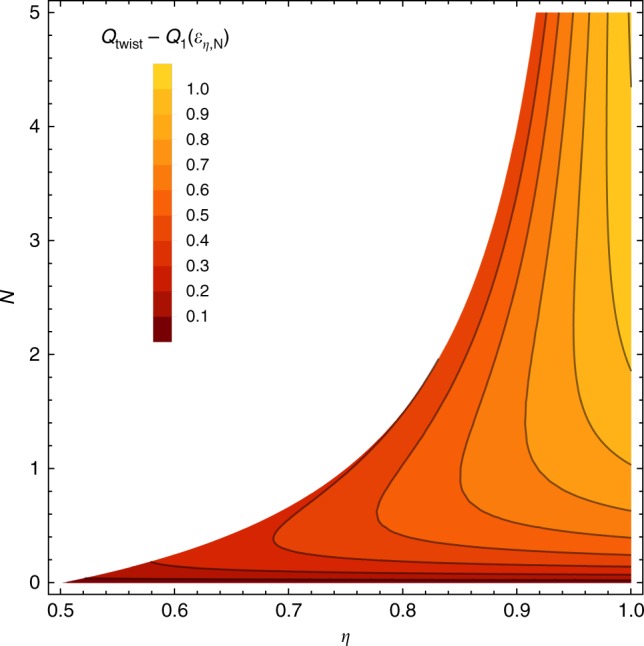
Best-approximation accuracy of the quantum capacity of a bosonic thermal attenuator. Contour plot of the difference between *Q*_twist_ of Eq. (40), i.e., the twisted decomposition upper bound of the quantum capacity of the bosonic Gaussian thermal attenuator, and the lower bound $Q_1\left( {{\cal E}_{\eta ,N}} \right)$ of Eq. (18), i.e., the single-letter capacity of the channel, as a function of the attenuation parameter *η* ∈ [0.5, 1] and noise values *N* ∈ [0, 5]. The white region corresponds to zero capacity. Observe that the approximation is tight in the small-noise region and, at higher values of noise, in the strong-attenuation region. Note that, as shown in Fig. 3b, in the opposite regime of high values of *N* and *η*, the quantum capacity is better upper bounded by (19)

One can easily check that this last bound is always better than the one derived in Eq. (33) and the bound *Q*_SWAT_ of Eq. (19). Moreover, for sufficiently small *η* or for sufficiently small *N*, it outperforms also the bound *Q*_PLOB_ of Eq. (19), see Fig. 3. By combining our result, *Q*_twist_, with *Q*_PLOB_ and with the lower bound *Q*_1_(Φ_*η*,*N*_), the quantum capacity is now constrained within a very small uncertainty window. Figure 4 shows the tiny gap existing between our new upper bound (40) based on the twisted decomposition and the lower bound (18).

## Discussion

In this article we computed some upper bounds on the quantum capacity of thermal attenuator channels, making use of an extended channel whose degradability properties are preserved when the environment has non-zero mean energy. This method gave interesting bounds in both the qubit and bosonic case, which are tight in the low temperature limit. Our method is quite general since it can be applied to any weakly degradable channel that admits a physical dilation with a mixed environmental state, not necessarily thermal. For example, one can apply this method straightforwardly to the bosonic thermal amplifier, though in this case the previously known upper bound^42^ is very tight and cannot be improved in this way. The second method we employed is less general, since it relies on a specific decomposition of thermal attenuators, but provides a better upper bound. Moreover, the twisted decomposition of Gaussian channels that we introduced in this work is an interesting result in itself which could find application in other contexts.

Our methods are of general interest for computing also other information capacities. Indeed, the channels that we introduce, i.e., the extended channel and those constituting the twisted decomposition, are by construction less noisy than the TA, in the sense that the latter can be obtained by any of the former via concatenation with another channel. This key property allows in principle to upper bound any information capacity of a TA with that of any of the channels we introduced. Of course, this may turn out to be very difficult in practice, depending on the kind of capacity that we are interested in. For example, a bound on the private capacity seems straightforward to derive, whereas it would require more efforts to bound the two-way and the strong-converse quantum capacities, the former because of the lack of a closed expression and the latter because of the difficult regularization involved in its formula. For these reasons, we believe that the channels we introduced are worth investigating also in the context of bounding other information capacities and may provide further interesting results.

Combining the results of this work with other previously known bounds, we can now estimate the value of the quantum capacity of thermal attenuator channels up to corrections, which are irrelevant for most practical purposes.

## Methods

### Computation of the upper bound for Qubit TA

The capacity of the extended qubit attenuator ${\mathrm{\Gamma }}_{\eta ,N}^{\mathrm{e}}$ can be computed by maximizing the coherent information of the channel, which is additive, as discussed in the main text:

41 $$\begin{array}{*{20}{l}} {Q_1\left( {{\mathrm{\Gamma }}_{\eta ,N}^{\mathrm{e}}} \right)} \hfill & = \hfill & {\mathop {{{\mathrm{max}}}}\limits_{\hat \rho } \left[ {S\left( {{\mathrm{\Gamma }}_{\eta ,N}^{\mathrm{e}}\left( {\hat \rho } \right)} \right) - S\left( {{\tilde{\mathrm \Gamma }}_{\eta ,N}^{\mathrm{e}}\left( {\hat \rho } \right)} \right)} \right]} \hfill \\ {} \hfill & = \hfill & {\mathop {{{\mathrm{max}}}}\limits_{p,\gamma } J_{\eta ,N}(p,\gamma )} \hfill \end{array}$$

and we have defined for simplicity of notation

42 $$J_{\eta ,N}(p,\gamma ) = J\left( {\hat \rho = \left[ {\begin{array}{*{20}{c}} {1 - p} & \gamma \\ {\gamma ^ \ast } & p \end{array}} \right],{\mathrm{\Gamma }}_{\eta ,N}^{\mathrm{e}}} \right),$$

depending on the parameters *p*, *γ* of the input qubit and on the channel parameters *η*, *N*. Recall that the extended channel ${\mathrm{\Gamma }}_{\eta ,N}^{\mathrm{e}}$ maps states of the system A to states of the joint system BE’ that includes the purifying part of the environment. Conversely, its complementary ${\tilde{\mathrm \Gamma }}_{\eta ,N}^{\mathrm{e}}$ maps states of A to states of the interacting part of the environment F. Therefore to compute their entropies we start by writing the joint state of the system AEE’, in the basis $\left\{ {\left| 0 \right\rangle ,\left| 1 \right\rangle } \right\}^{ \otimes 3}$:

43 $${\hat \rho _{{\mathrm{AEE}}\prime } = \left( {\begin{array}{*{20}{c}} {(1 - N)(1 - p)} & 0 & 0 & {\sqrt {(1 - N)N} (1 - p)} & {\gamma (1 - N)} & 0 & 0 & {\sqrt {(1 - N)N} \gamma } \\ 0 & 0 & 0 & 0 & 0 & 0 & 0 & 0 \\ 0 & 0 & 0 & 0 & 0 & 0 & 0 & 0 \\ {\sqrt {(1 - N)N} (1 - p)} & 0 & 0 & {N(1 - p)} & {\sqrt {(1 - N)N} \gamma } & 0 & 0 & {N\gamma } \\ {(1 - N)\gamma ^ \ast } & 0 & 0 & {\sqrt {(1 - N)N} \gamma ^ \ast } & {p - Np} & 0 & 0 & {\sqrt {(1 - N)N} p} \\ 0 & 0 & 0 & 0 & 0 & 0 & 0 & 0 \\ 0 & 0 & 0 & 0 & 0 & 0 & 0 & 0 \\ {\sqrt {(1 - N)N} \gamma ^ \ast } & 0 & 0 & {N\gamma ^ \ast } & {\sqrt {(1 - N)N} p} & 0 & 0 & {Np} \\ {} & {} & {} & {} & {} & {} & {} & {} \end{array}} \right).}$$

Next, we compute its evolved $\hat \rho _{{\mathrm{BFE}}\prime }$ under the action of ${\cal U}_{{\mathrm{AE}} \to {\mathrm{BF}}}^{(\eta )} \otimes {\bf{I}}_{{\mathrm{E}}\prime }$ and the marginals with respect to the bipartition BE′-F, which correspond to the output states of the two channels:

44 $$	{\mathrm{\Gamma }}_{\eta ,N}^{\mathrm{e}}\left( {\hat \rho } \right) = \\ 	\left( {\begin{array}{*{20}{c}} {(1 - N)(1 - p\eta )} & {\sqrt {(1 - N)N(1 - \eta )\eta } \gamma ^ \ast } & {\gamma (1 - N)\sqrt \eta } & {(2p - 1)\sqrt {(1 - N)N(1 - \eta )} } \\ {\gamma \sqrt {(1 - N)N(1 - \eta )\eta } } & {N(1 - p)\eta } & 0 & {N\gamma \sqrt \eta } \\ {(1 - N)\sqrt \eta \gamma ^ \ast } & 0 & {p(1 - N)\eta } & { - \sqrt {(1 - N)N(1 - \eta )\eta } \gamma ^ \ast } \\ {(2p - 1)\sqrt {(1 - N)N(1 - \eta )} } & {N\sqrt \eta \gamma ^ \ast } & { - \gamma \sqrt {(1 - N)N(1 - \eta )\eta } } & {(p - 1)\eta N + N} \\ {} & {} & {} & {} \end{array}} \right),\\ 	{\tilde{\mathrm \Gamma }}_{\eta ,N}^{\mathrm{e}}\left( {\hat \rho } \right) = \left( {\begin{array}{*{20}{c}} { - p(1 - \eta ) - N\eta + 1} & {(1 - 2N)\gamma \sqrt {1 - \eta } } \\ {(1 - 2N)\sqrt {1 - \eta } \gamma ^ \ast } & { - \eta p + p + N\eta } \\ {} & {} \end{array}} \right).$$

The entropies of these states can be computed numerically and it can be checked that they are invariant under phase-flip, i.e., *γ* → −*γ*. Hence we obtain an expression of the coherent information that is an even function of *γ* and write, following^5^:

45 $$J_{\eta ,N}(p,\gamma ) = \frac{{J_{\eta ,N}(p,\gamma ) + J_{\eta ,N}(p, - \gamma )}}{2} \le J_{\eta ,N}(p,0),$$

where the inequality follows from the concavity of the mutual information as a function of the input state, which holds since the channel is degradable^50^. Hence we have restricted the optimization to diagonal states in the chosen basis, i.e., on the single parameter *p* for fixed *η*, *N*:

46 $$Q_1\left( {{\mathrm{\Gamma }}_{\eta ,N}^{\mathrm{e}}} \right) = \mathop {{{\mathrm{max}}}}\limits_p J_{\eta ,N}(p,0),$$

for *η* > 1/2 and 0 otherwise. The latter expression can be easily solved numerically, as shown by the plots in the main text.

### Twisted decomposition of phase-insensitive Gaussian channels

Here we generalize the twisted decomposition introduced in the main text for bosonic thermal attenuators to the more general class of phase-insensitive Gaussian channels ${\cal G}_{\tau ,y}$, defined by the following action on the first and second moments of single-mode Gaussian states^37^:

47 $${\boldsymbol{m}}\mathop{\longrightarrow}\limits^{{{\cal G}\tau ,y}}{\boldsymbol{m}}\prime = \sqrt \tau {\boldsymbol{m}},$$

48 $$V\mathop{\longrightarrow}\limits^{{{\cal G}\tau ,y}}V\prime = \tau V + y{\mathbf{1}}_2,$$

where *τ* ≥ 0 is a generalized transmissivity and $y \ge \left| {1 - \tau } \right|$ is a noise parameter^27^. This family includes the thermal attenuator for 0 ≤ *τ* < 1, the thermal amplifier for *τ* > 1, and the additive Gaussian noise channel for *τ* = 1. If $y = \left| {1 - \tau } \right|$, the channel introduces the minimum noise allowed by quantum mechanics and is said to be quantum-limited. On the other hand, it can be shown^27,53^ that a phase-insensitive Gaussian channel is entanglement-breaking if and only if *y* ≥ 1 + *τ*, which determines a noise threshold above which the channel has trivially zero quantum capacity. Below the entanglement-breaking threshold, the following decomposition holds.*Lemma 2*. Every phase-insensitive Gaussian channel ${\cal G}_{\tau ,y}$ which is not entanglement-breaking (*y* < 1 + *τ*), can be decomposed as a quantum-limited amplifier followed by a quantum-limited attenuator:

49 $${\cal G}_{\tau ,y} = {\cal G}_{\eta \prime ,1 - \eta \prime } \circ {\cal G}_{\kappa \prime ,\kappa \prime - 1} = {\cal E}_{\eta \prime ,0} \circ {\cal A}_{\kappa {\prime},0},$$

with attenuation and gain coefficients given by

50 $$\eta \prime = (1 + \tau - y){\mathrm{/}}2,\kappa \prime = \tau {\mathrm{/}}\eta \prime .$$

The proof follows by direct substitution of the parameters (50) into (49) and from the application of Eqs. (47) and (48). Moreover, the hypothesis *y* < 1 + *τ* is necessary since it ensures the positivity of both the attenuation and the gain parameters *η*′ and *κ*′.

## Data Availability

No datasets were generated or analyzed during the current study.
