# Author Correction: Narrow bounds for the quantum capacity of thermal attenuators

**DOI:** 10.1038/s41467-019-08338-3

**Published:** 2019-01-15

**Authors:** Matteo Rosati, Andrea Mari, Vittorio Giovannetti

**Affiliations:** 1 Física Teòrica: Informació i Fenòmens Quàntics, Departament de Física, Universitat Autònoma de Barcelona, 08193 Bellaterra, Spain; 2 NEST, Scuola Normale Superiore and Istituto Nanoscienze-CNR, 56127 Pisa, Italy

Correction to: *Nature Communications*; 10.1038/s41467-018-06848-0; published online 18 October 2018

The original version of this Article contained an error in Equation (40). The numerator of the fraction inside the logarithm was missing an overall minus sign, and incorrectly read:

$$Q\left( {{\mathrm{\Phi }}_{\eta ,N}} \right) \le Q_{{\mathrm{twist}}} = \max \left\{ {0,\log _2\frac{{N\left( {1 - \eta } \right) - \eta }}{{\left( {1 + N} \right)\left( {1 - \eta } \right)}}} \right\}$$

The correct form of Equation (40) is:

$$Q\left( {{\mathrm{\Phi }}_{\eta ,N}} \right) \le Q_{{\mathrm{twist}}} = \max \left\{ {0,\log _2\frac{{\eta - N\left( {1 - \eta } \right)}}{{\left( {1 + N} \right)\left( {1 - \eta } \right)}}} \right\}$$

This error has been corrected in both the PDF and HTML versions of the Article.

